# Regional biogeography of microbiota composition in the Chagas disease vector *Rhodnius pallescens*

**DOI:** 10.1186/s13071-019-3761-8

**Published:** 2019-10-29

**Authors:** Troy J. Kieran, Kaylee M. H. Arnold, Jesse C. Thomas, Christina P. Varian, Azael Saldaña, Jose E. Calzada, Travis C. Glenn, Nicole L. Gottdenker

**Affiliations:** 1 Department of Environmental Health Science, College of Public Health, University of Georgia, Athens, GA USA; 2 Odum School of Ecology, University of Georgia, Athens, GA USA; 3 Center for the Ecology of Infectious Diseases, University of Georgia, Athens, GA USA; 4 Department of Veterinary Pathology, College of Veterinary Medicine, University of Georgia, Athens, GA USA; 5 Instituto Conmemorativo Gorgas de Estudios de la Salud (ICGES), Panama City, Panama; 6 Institute of Bioinformatics, University of Georgia, Athens, GA USA

**Keywords:** Triatominae, Triatomine, *16S* rRNA, Microbiome, Metabarcoding, *Wolbachia*, *Attalea* palms, *Trypanosoma*, Panama, Vector-borne disease

## Abstract

**Background:**

Triatomine bugs are vectors of the protozoan parasite *Trypanosoma cruzi*, which causes Chagas disease. *Rhodnius pallescens* is a major vector of Chagas disease in Panama. Understanding the microbial ecology of disease vectors is important in the development of vector management strategies that target vector survival and fitness. In this study we examined the whole-body microbial composition of *R. pallescens* from three locations in Panama.

**Methods:**

We collected 89 *R. pallescens* specimens using Noireau traps in *Attalea butyracea* palms. We then extracted total DNA from whole-bodies of specimens and amplified bacterial microbiota using *16S* rRNA metabarcoding PCR. The *16S* libraries were sequenced on an Illumina MiSeq and analyzed using QIIME2 software.

**Results:**

We found Proteobacteria, Actinobacteria, Bacteroidetes and Firmicutes to be the most abundant bacterial phyla across all samples. Geographical location showed the largest difference in microbial composition with northern Veraguas Province having the most diversity and Panama Oeste Province localities being most similar to each other. *Wolbachia* was detected in high abundance (48–72%) at Panama Oeste area localities with a complete absence of detection in Veraguas Province. No significant differences in microbial composition were detected between triatomine age class, primary blood meal source, or *T. cruzi* infection status.

**Conclusions:**

We found biogeographical regions differ in microbial composition among *R. pallescens* populations in Panama. While overall the microbiota has bacterial taxa consistent with previous studies in triatomine microbial ecology, locality differences are an important observation for future studies. Geographical heterogeneity in microbiomes of vectors is an important consideration for future developments that leverage microbiomes for disease control.

## Background

Insect microbiota are composed of a wide variety of microbial species [1, 2] that serve as commensals, pathogens, or have mutualistic benefits that impact the reproduction, nutrition, and immune systems of the insect host [1–4]. The symbiotic relationship between an insect disease vector and its microbiota can have an important influence on the competence and transmission potential of human diseases [4, 5], increasing or decreasing pathogen transmission from vector to host [6], including in blood-feeding species [3, 7]. Insect microbiota research can lead to improved methods of vector control [8–13], with substantial research in some mosquito species, but limited research in many other vector species, often conflicting results, leaves many questions unanswered [6]. Vector life stage, distribution, species, methods/sampling strategies and environment (e.g. habitat type or geographical region) may influence vector microbiota [6, 14–25].

In this study, we evaluated patterns of whole-body microbiota of a Chagas disease vector. Chagas disease, caused by the kinetoplastid protozoan parasite *Trypanosoma cruzi*, is transmitted between a wide range of potential mammalian hosts and humans by hematophagous (blood-feeding) triatomine insect vectors. Despite widespread control programmes, Chagas disease remains a significant health threat to millions of inhabitants in Latin America, particularly those that live in poverty [26]. The idea of using bacterial symbionts of triatomine bugs to control Chagas disease has long been proposed [27]. Recent studies describe microbial community composition within triatomines [23, 28–33], including *R. pallescens* from Colombia [22] and Panama [34], and a sister species, *R. prolixus* [35]. Studies thus far describe triatomine microbiota as having low complexity in terms of diversity and species-specific patterns [23, 29, 30], yet the microbiota for many taxa remain to be studied.

Infection of triatomines with trypanosomes has been associated with reduction in gut microbial diversity [28, 35], and blood meal identity may influence composition of the predominant bacterial taxa [30, 31]. However, other important comparisons among triatomines are lacking, such as differences between location and habitat type. Microbial composition variation between different geographical locations has been observed in ticks [15, 18, 20, 24, 25] and with mixed observations in mosquitoes [16, 36]. Habitat has also been shown to be a main driver of microbial species composition in mosquitoes [37, 38].

There are more than 150 species of triatomines, with different distributions, habitat requirements and life histories that can impact microbiota, or microbiota that can affect vectorial capacity. This complexity requires extensive research into different triatomine microbiomes. Currently, we still lack basic microbial community composition descriptions for many triatomine species and these large gaps in our knowledge make informed research for vector biocontrol difficult. Therefore, advancing research in triatomine microbiota is crucial for gaining a better understanding of *T. cruzi* infection, triatomine vector capacity, and developmental biology.

*Rhodnius pallescens* is the main vector *Trypanosoma cruzi* and *T. rangeli* in Panama [39] where they are widely distributed throughout Panama and into the neighboring countries of Costa Rica and Colombia. *Rhodnius pallescens* is commonly found and associated with *Attalea butyracea* palms [40, 41] that are common and widely distributed across many different habitats in Panama [40, 41]. The sylvatic behavior has complicated control efforts [42]. New endemic regions are still being described [43–45] and a darker chromatic variation of *R. pallescens* associated with distinct genetic groups of *T. cruzi* and *T. rangeli* has also been found recently in Santa Fe District, Panama [44].

Here, we describe the whole-body bacterial microbiota of wild-caught *R. pallescens* from three separate geographical locations in Panama. We used the entire triatomine body to encompass all potential microbial taxa relevant to *R. pallescens* that could affect their fitness and survival as a benchmark for future studies of localized anatomy microbiota. We hypothesize that both habitat type and geographical location will be associated with differences in whole-body microbiota composition similar to previous findings in other vectors [15, 16, 18, 20, 24, 25, 37, 38]. We further hypothesize that complex environments, such as forest patches, will be associated with a more diverse microbiota composition in insects in more homogenous environments (i.e. cattle pastures), as previous studies in insects show that environmental diversity leads to increased microbial diversity [46–48]. We used Illumina *16S* rRNA amplicon sequencing to characterize and evaluate the bacterial microbiota of *R. pallescens* between different locations and habitats, comparing infection status, age class, and primary blood-meal source to evaluate a range of variables that may be associated with whole-body bacterial community composition.

## Methods

### Sample collection and DNA extraction

**Fig. 1:**
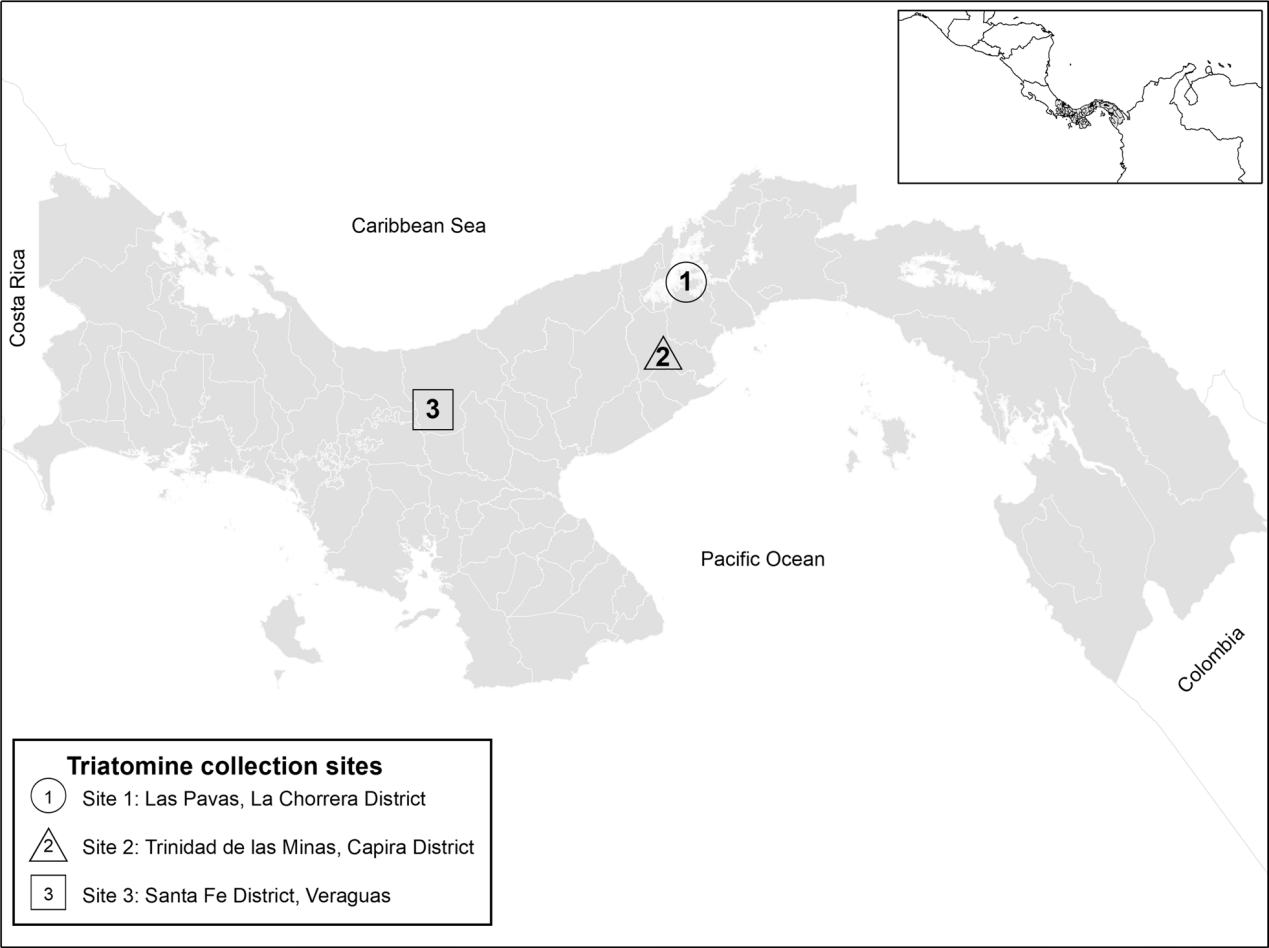
Map of Panama showing the locations of the three collection sites

All *R. pallescens* evaluated specimens (*n* = 89) were collected in Panama, Central America using Noireau traps [49] in *Attalea butyracea* palms (the main habitat of this species) and placed directly in 95% molecular grade ethanol before use. We sampled from a total of 8 palms, in three habitats (pasture, peridomestic and peridomestic-forest), from three geographic locations in lowland moist tropical forest (Las Pavas and Trinidad de las Minas) and moist tropical forest (Santa Fe and Veraguas) (Fig. 1). We consider peridomestic to be home yards or areas within 100 m of a dwelling and peridomestic-forest to be patches of regenerated forest within a peridomestic landscape matrix. Samples from Las Pavas, La Chorrera District (9.104167° N, 79.885833° W) (*n* = 27 from two habitats) and Trinidad de las Minas, Capira District (8.775556° N, 79.995833° W) (*n* = 32, from one habitat) were from a previous study examining blood meals [50]. We further collected 30 samples from four sites comprising three habitats located in Santa Fe District, Veraguas (8.509232° N, 81.077800° W) from 8–11 July 2017. The Santa Fe region has recently been described as a new endemic focus for Chagas disease in Panama, where a dark morph of *R. pallescens* predominates [43, 44]. All specimens were nymphs, primarily N3 and below (92%, 95% CI: 84.4–96.39%), with the exception of one male from Trinidad de las Minas (Additional file 1: Table S1). DNA was extracted from whole specimens following Kieran et al. [50]. Briefly, samples were macerated and digested overnight in digest buffer with proteinase K and extracted with phenol-chloroform-isoamyl alcohol. Extractions were reconstituted in TLE buffer (10 mM Tris, pH 8; 0.1 mM EDTA), and impurities were removed with Sera-Mag SpeedBeads™ (Thermo Fisher Scientific, Waltham, MA, USA; [51]) with a final reconstitution in 30 µl TLE.

### DNA amplification and sequencing

We amplified bacterial *16S* rRNA DNA using the S-D-Bact-0341-b-S-17 (5′-CCT ACG GGN GGC WGC AG-3′) forward and S-D-Bact-0785-a-A-21 (5′-GAC TAC HVG GGT ATC TAA TCC-3′) reverse primer pair [52] to which we added modifications following previous studies [50, 53, 54]. We added Illumina TruSeq sequences to the 5′-end of the forward (Read 1) and reverse (Read 2) primer creating fusion primers. We synthesized 8 forward and 12 reverse fusion primers, each with a unique variable length (5–8 bp) index sequence between the *16S* and TruSeq sequences. We then performed two rounds of PCR. For the first-round we performed replicate PCRs using 12.5 μl reactions of KAPA HiFi HotStart Kits (Kapa Biosystems, Wilmington, MA, USA) consisting of 2.5 μl of 5× buffer, 0.375 μl of 10 mM dNTPs, 0.25 μl hot start Taq, 5.4 μl molecular grade water, 1 μl of 5 μM forward primer, 1 μl of 5 μM reverse primer, and 2 μl of DNA. The ranges of DNA sample concentrations were from 10 ng/μl to 60 ng/μl. Each DNA sample had a unique primer-index combination with the following thermocycler conditions: 98 °C for 3 min, followed by 30 cycles at 95 °C for 30 s, 63 °C for 1 min, 72 °C for 1 min and a final extension at 72 °C for 5 min. Amplification success was verified on a 1.5% agarose gel.

Amplicons were pooled in equal concentrations and cleaned using a 1:1 ratio of SPRI-beads and reconstituted in 25 μl TLE. The second-round PCR primers consisted of Illumina TruSeqHT compatible 8 nt indexed primers [55]. We used 25 μl reaction of KAPA HiFi HotStart Kits using 5 μl of 5× Buffer, 0.75 μl of 10 mM dNTPs, 0.5 μl HotStart, 3.75 μl molecular grade water, 2.5 μl of 5 μM forward primer, 2.5 μl 5 μM reverse primer, and 10 μl of *16S* amplicon pool. We performed two replicate PCRs with the following thermocycler conditions: 98 °C for 2 min, followed by 10 cycles at 98 °C for 30 s, 60 °C for 30 s, 72 °C for 30 s and a final extension at 72 °C for 5 min. Library product was cleaned with Sera-Mag SpeedBeads™ (1:1 ratio) and pooled with other uniquely indexed samples prior to sequencing.

For blood-meal source data, we used previous data from Kieran et al. [50] and for newly collected samples, we amplified *12S* rRNA gene following Kieran et al. [50]. All libraries were sent to the Georgia Genomics and Bioinformatics Core (http://dna.uga.edu) for sequencing on an Illumina MiSeq using a v3 PE300 kit (Illumina, San Diego, CA, USA). We also screened for the presence of *T. cruzi* and *T. rangeli* amplifying telomeric kinetoplastid DNA with Tc189 and Tr primers [56]. Samples were also verified for *T. cruzi* using 121/122 primers targeting the kinetoplastid minicircle [57]. Amplification success was verified on a 1.5% agarose gel.

### Data processing and analysis

We demultiplexed the amplicon indices using Mr. Demuxy 1.2.0 (https://pypi.org/project/Mr_Demuxy/) and resulting fastq files were imported into Geneious 10.0.1 [58]; (https://www.geneious.com) where we trimmed primers, paired and merged the reads using FLASH [59]. Subsequent data were exported as fastq files for importation into Qiime2 [60]. The quality of the sequences was checked and filtered using QIIME2 v. 2018.8 plugin DADA2 [61] and chimeric sequences were removed. The remaining forward sequences were truncated to a final length of 292 bp and the reverse sequences were truncated to a final length of 240 bp. Amplicon sequence variants (ASV) were analyzed using the q2-diversity Qiime2 plugin to calculate multiple alpha diversity metrics, including Shannonʼs index H’, Simpsonʼs index D_s_, Chao1, Faith’s phylogenetic diversity, and observed-ASV’s.

The Qiime2 plugin q2-phylogeny was used to complete a multiple sequence alignment to reconstruct rooted and unrooted phylogenetic trees from the filtered alignment based on maximum-likelihood approximation with FastTree 2 [62]. Alpha and beta diversity metrics were assigned using the q2-diversity Qiime2 plugin. An alpha rarefaction was used to evaluate sampling depth, and the data was rarefied at 1000 sequences per sample, removing two samples and retaining 87 samples for final analyses.

The Qiime2 plugin q2-feature-classifier was used to align the sequences against the Greengenes 13.8 database [63]. OTUs were identified from phyla down to the genus level, we removed archaea, chloroplasts, mitochondria, not available (NAs), and uncharacterized taxa at the kingdom level.

Alpha diversity (species diversity) was calculated using Shannonʼs (species richness), Simpson’s (evenness or relative abundance), and Chao1 (estimate of diversity from abundance) diversity metrics. To compare alpha diversities from individuals across location, habitat type, and infection status, a one‐way analysis of variance (ANOVA) and *post‐hoc* Tukeyʼs honest significant difference (HSD) tests for multiple comparisons were performed to evaluate differences in taxonomic abundance and alpha diversities. A *P*-value less than 0.05 was considered statistically significant.

Beta diversity (compositional variation) was calculated for the whole-body microbiota comparison between triatomines across location, habitat type, and infection status using Bray-Curtis dissimilarity. Bray-Curtis is based on shared OTU counts between individuals. Finally, we used non-metric multidimensional scaling (nMDS) [64] to visualize differences between the microbial communities, and a permutational MANOVA for hypothesis testing [65]. All diversity analyses and visualizations were conducted using qiime2 artifact outputs in R (v. 3.5.1) and with the packages *phyloseq* [66], *vegan* [67], *dplyr* [68], *ggplot2* [69] and *metacoder* [70]. To further assess our findings of a location effect we removed older age classes (N4, N5, Adult) and repeated the analysis (*n* = 80).

## Results

### *16S* rRNA sequences and classification of entire microbiota community

**Fig. 2:**
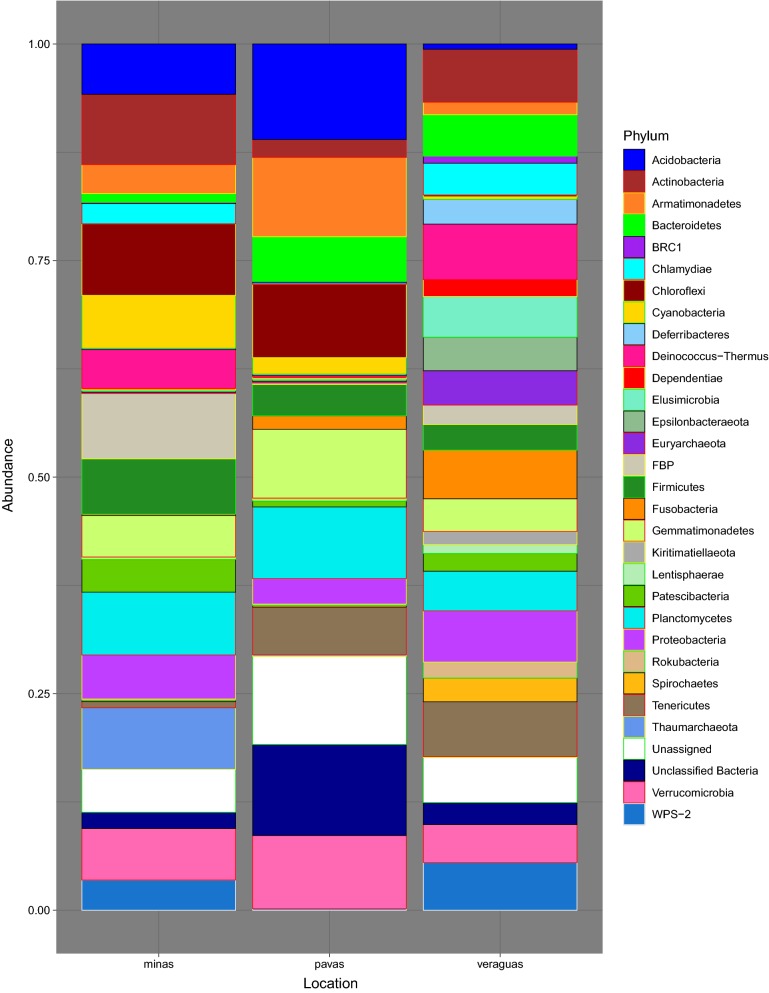
Taxonomic composition at the phylum level by location

**Fig. 3:**
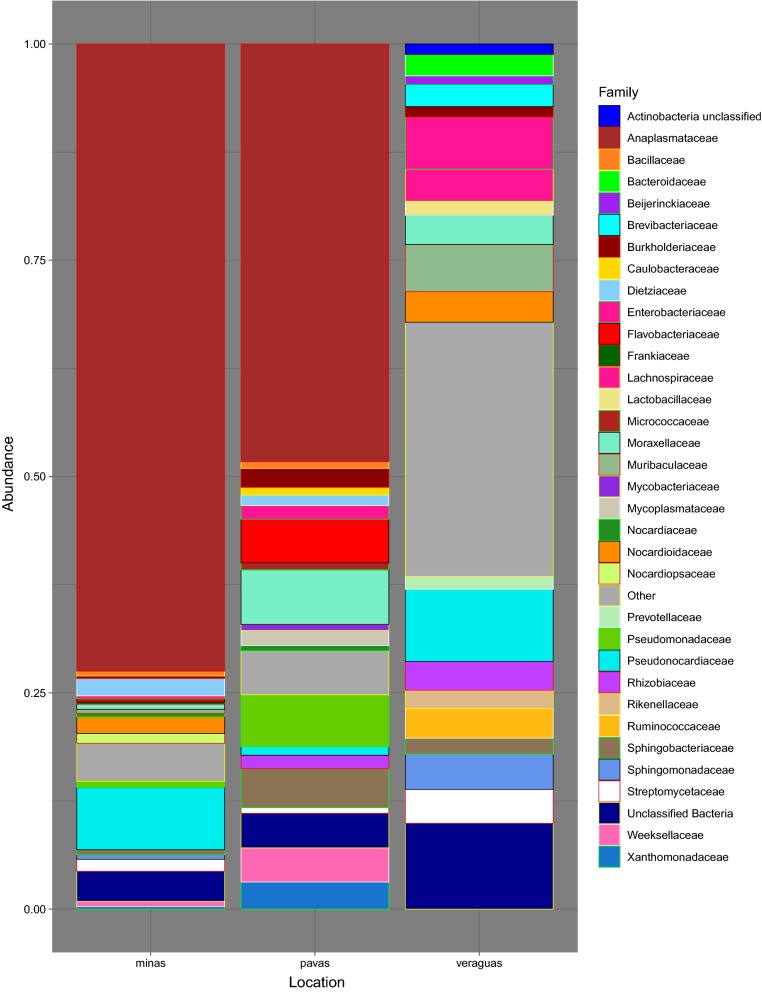
Top 20 taxonomic composition per location at the family level

**Fig. 4:**
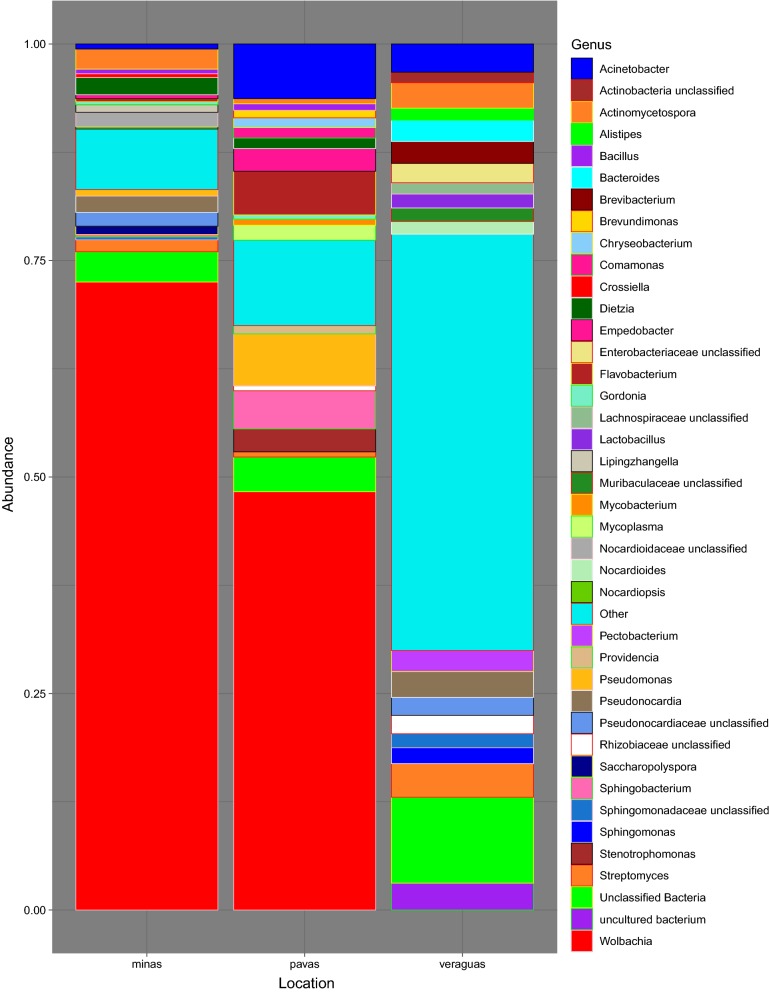
Top 20 taxonomic composition per location at the genus level

We obtained a total of 4,995,733 *16S* rRNA V3–V4 region sequences from 101 samples, including the negative controls of molecular grade water and positive controls of *E. coli*. After quality filtering and the exclusion of 14 samples due to low read numbers, the number of sequences obtained per sample ranged from 1000 to 34,792 reads, with a mean frequency of 9811.63. The total number of OTUs within the 87 final samples was 4033, with the top 4 phyla consisting of Proteobacteria (60.67%), Actinobacteria (16.93%), Bacteroidetes (9.55%) and Firmicutes (4.11%) out of the total phyla present in the dataset. *Rhodnius pallescens*, in Las Pavas and Trinidad de las Minas respectively, is primarily composed of Proteobacteria (71.63% and 76.11%, respectively), Actinobacteria (6.13% and 15.88%, respectively), and Bacteroidetes (13.98% and 2.66%, respectively) (Fig. 2, Additional file 2: Table S2, Additional file 3: Figure S1). This contrasts with specimens from northern Veraguas with the most abundant phylum shifting from Proteobacteria (26.43%) to Actinobacteria (27.56%) and introducing more Firmicutes (11.48%). At the family-level (Fig. 3, Additional file 3: Figure S1), the top 3 taxa overall were *Anaplasmataceae* (45.76%), *Pseudonocardiaceae* (6.04%), *Moraxellaceae* (2.77%), although these relative proportions differ by location (Additional file 4: Table S3, Additional file 3: Figure S1). Most notably, as seen in Fig. 3, *Anaplasmataceae* was the dominant family throughout samples from Las Pavas (48.30%) and Trinidad de las Minas (72.51%) (Fig. 4, Additional file 5: Table S4, Additional file 3: Figure S1) but is not present within samples collected in northern Veraguas. The high abundance of *Anaplasmataceae* was due to a single genus, *Wolbachia* spp., comprising greater than 70% and 42% of the composition in more than half the specimens from Trinidad de las Minas and Las Pavas, respectively (Additional file 6: Table S5). No differences in microbial composition were detected between triatomine age class (Shannon, *F*_(5, 85)_ = 1.07, *P* > 0.09) or primary blood-meal source (Shannon, F_(9, 77)_ = 1.07, *P* > 0.38).

### Infection rates

*Trypanosma cruzi* and *T. rangeli* were detected in sampled vectors at all locations (Additional file 1: Table S1). Rates of positive *T. cruzi* infection were 7.41% (2/27, 95% CI: 0.96–24.47%) at Las Pavas (*n* palms = 2), 75% (24/32, 95% CI: 57.67–86.97%) at Trinidad de las Minas (*n* palms = 2), and 46.67% (14/30, 95% CI: 30.23–63.46%) at Santa Fe District, Veraguas (*n* palms = 4). When examining *T. cruzi* infection by habitat we find rates of 12.5% (1/8, 95% CI: 0.11–49.2%) in Las Pavas pasture compared to 50% (8/16, 95% CI: 28–72%) in Veraguas pasture. In peridomestic areas, rates varied from 5.26% (1/19, 95% CI: < 0.01–26.48%) in Las Pavas and 42.85% (3/7, 95% CI: 15.75–75.02%) in Veraguas. Peridomestic-forest habitat in Veraguas had an infection rate of 42.85% (3/7, 95% CI: 15.75–75.02%).

For *T. rangeli*, positive detection rates were 66.67% (18/27, 95% CI: 47.71–81.47%) in Las Pavas, 78.13% (25/32, 95% CI: 60.96–89.27%) in Trinidad de las Minas, and 30% (9/30, 95% CI: 16.52–48.02) in Veraguas. There were low rates of *T. rangeli* infection in both Las Pavas (0%) and Veraguas (6.25%, 1/16, 95% CI: < 0.01–30.31%) pastures, while peridomestic areas were high (94.74%, 18/19, 95% CI: 73.52– > 99.99%) to moderate (28.57%, 2/7, 95% CI: 7.56–64.76%) at each respective location. Veraguas peridomestic-forest infection rate was 85.7% (6/7, 95% CI: 46.65–99.47).

There was an overall rate of coinfections of both trypanosomes of 31.46% (28/89, 95% CI: 22.72–41.73%). Coinfections were less abundant at the lower infection sites of Las Pavas (3.7%, 95% CI: < 0.01–19.8%) and Veraguas (13.33%, 95% CI: 4.7–30.3%) compared to Trinidad de las Minas which had a higher coinfection rate (71.88%, 95% CI: 54.46–84.6%). At the habitat level, coinfection rates varied from non-existent (0%) in pastures, to low (5.26%, 1/19, 95% CI: < 0.01–26.48%) and moderate (28.57%, 2/7, 95% CI: 7.56–64.76) in peridomestic areas for Las Pavas and Veraguas, respectively. Coinfection rate for peridomestic-forest was the same as peridomestic areas in Veraguas.

### Location, habitat type, and infection status on microbial composition

#### Alpha diversity

Alpha richness between locations (Additional file 7: Figure S2) was significantly different using three different diversity metrics (ANOVA, *F*_(2, 85)_ = 12.16, *P* < 0.001; Shannon’s index H’ ANOVA, *F*_(2, 85)_ = 11.7, *P* < 0.001; Chao1 ANOVA, *F*_(2, 85)_ = 12.04, *P* < 0.001) even when older aged individuals were removed (ANOVA, *F*_(2, 77)_ = 10.89, *P* < 0.001; Shannon’s index H’ ANOVA, *F*_(2, 77)_ = 11.38, *P* < 0.001; Chao1 ANOVA, *F*_(2, 77)_ = 10.78, *P* < 0.001). Individuals from Veraguas had significantly greater alpha richness when compared to individuals from Trinidad de las Minas (Chao1, Shannon, TukeyHSD, *P* < 0.0001; Simpson, Tukey HSD *P* = 0.008) and Las Pavas (TukeyHSD, *P* < 0.0004, Chao1, TukeyHSD, *P* = 0.0004; Shannon, TukeyHSD, *P* = 0.0096). These results remained the same for all metrics when older age classes were removed (*P* < 0.001 for Trinidad de las Minas; *P* < 0.0093 for Las Pavas). Alpha richness across habitat type (Additional file 8: Figure S3) showed significance for one metric (Shannon, TukeyHSD, *F*_(2, 85)_ = 4.72, *P* = 0.011; older classes removed *F*_(2, 77)_ = 2.24, *P* = 0.006) between peridomestic and peridomestic-forest types (*P* = 0.009; older classes removed *P* = 0.004). This trend holds when examining habitat across the Veraguas site (*P* = 0.001). *Trypanosoma cruzi* infection status (Additional file 9: Figure S4), however, was not significantly different (Simpson, *F*_(2, 78)_ = 3.54, *P* > 0.063).

#### Beta diversity

All PERMANOVA results are reported using Bray-Curtis dissimilarity indices. The community differences (beta diversity) of triatomine microbiota showed significant differences at pasture sites across Trinidad de las Minas and Veraguas (PERMANOVA, *F*_(1, 20)_ = 5.61, *P* = 0.001; Additional file 10: Figure S5) and at peridomestic sites across all three locations (PERMANOVA, *F*_(2, 61)_ = 12.09, *P* = 0.001; Additional file 11: Figure S6). Community differences across the three habitat types also showed some significant differences within Veraguas (PERMANOVA, *F*_(2, 25)_ = 1.41, *P* = 0.001; Additional file 12: Figure S7) and Las Pavas (PERMANOVA, *F*_(1, 25)_ = 3.96, *P* = 0.001; Additional file 13: Figure S8). There was no observed difference between pasture habitat composition between sites (PERMANOVA, *F*_(1, 22)_ = 1.88, *P* = 0.818). When examining *T. cruzi* infection status, the only significant differences in microbial composition between *T. cruzi*-positive and negative samples were observed at Las Pavas (PERMANOVA, *F*_(2, 24)_ = 1.31, *P* = 0.029; Additional file 14: Figure S9); however, positive infection was extremely low (2/27). Microbial composition and *T. cruzi* infection status was also significant between peridomestic habitats among all locations (PERMANOVA, *F*_(1, 56)_ = 2.21, *P* = 0.01, Additional file 15: Figure S10). Sites at Trinidad de las Minas (PERMANOVA, *F*_(2, 29)_ = 1.59, *P* = 0.65; Additional file 16: Figure S11) and Veraguas (PERMANOVA, *F*_(2, 25)_ = 1.29, *P* = 0.76; Additional file 17: Figure S12) did not show significant compositional difference between infected and non-infected samples. Results with the removal of older age classes were not substantially different. The lack of effect of older nymphal stages is further supported by multivariate PCA in Additional file 18: Figure S13.

## Discussion

Here, we characterized the bacterial microbiota of 87 wild individuals of the Chagas disease vector *Rhodnius pallescens* from three populations in Panama. We explored comparisons in composition between location, microhabitat, nymphal stage, *T. cruzi* infection, and blood-meal status. Overall, the microbiota of *R. pallescens* exhibited relatively low complexity in its bacterial composition which is consistent with other triatomine studies [23, 29, 30]. Proteobacteria has also been found to be the most abundant phylum in other vector species [1, 71–75] including the triatomines *R. neglectus*, *R. prolixus*, *Triatoma vitticeps*, *T. infestans*, *T. brasiliensis*, *T. pseudomaculata*, *Dipetalogaster maximus* and *Panstrongylus megistus* [29, 30, 73], while a predominance of Actinobacteria has been found previously in *R. pallescens* [22], both consistent with our findings.

Common bacterial genera found in other triatomines include *Burkholderia*, *Dietzia*, *Gordonia*, *Williamsia* [22, 30, 73], *Actinomycetospora*, *Arsenophonus*, *Corynebacterium*, *Rhodococcus*, *Staphylococcus* [23, 30, 31], and *Enterococcus*, *Enterobacteriaceae*, *Bacillus* [23, 33]. Of these only *Actinomycetospora* was one of the top 20 genera found across all studied sites (Additional file 5: Table S4). *Enterobacteriaceae* and *Bacillus* were found at all sites, but at much lower abundance (0.34–6.03% and 0.21–0.76%, respectively) than found by Waltmann et al. (36.7% and 2%, respectively) [33]; however, the present study differs in that we analyzed wild and not laboratory-reared samples. *Dietzia* and *Gordonia* were each in the top 20 taxa for Las Pavas and Trinidad de las Minas (Additional file 5: Table S4). *Arsenophonus* was detected with a very low abundance in only a single specimen from northern Veraguas Province. All other taxa were found across all sites, but at lower abundance (Additional file 19: Table S6) than in other studies.

High levels of Proteobacteria observed in specimens from Panama Oeste Province localities are due to the very high levels of *Wolbachia* sp. Specimens from northern Veraguas Province, interestingly, did not have any *Wolbachia* present. It has been estimated that *Wolbachia* infects 52% of all aquatic insect species [76] and can infect a high proportion of the number of individuals in a species [77]. However, while many arthropod species may be infected with *Wolbachia*, a majority of the individuals within a species may not be. In a comparative study, Sazama et al. [76] found that less than half of the individuals were infected in most (69%) *Wolbachia*-infected species. *Wolbachia* has been found previously in *Rhodnius* sp. [29, 34] and is common in hematophagous insects [78], but has not been found in other triatomines [29, 31, 73]. In triatomines [29] and sandflies [74, 79, 80], the role of *Wolbachia* remains unknown. In mosquitoes, *Wolbachia* can affect reproduction and insecticide resistance among others [7], thus creating opportunities for vector biocontrol. However, without further knowledge of the role of *Wolbachia* in triatomines, identifying microbes that can serve as effective control agents for triatomines requires further research. To this end, we need more characterized microbiomes under multiple environmental conditions, and functional analysis of microbial taxa.

In our study, geographical location was associated with differences between microbial communities of *R. pallescens*. This observation is most evident between the two most disparate geographical locations (northern Veraguas *vs* Panama Oeste localities). This observation may be the result of quite different environments between these locations. Veraguas Province is located in the highlands of the western isthmus of Panama where the climate is cooler (mean temperature of 21 °C) and more humid with mountainous topography. In the two Panama Oeste area locations (Las Pavas and Trinidad de las Minas) the topography is flatter with warmer temperatures (mean 27 °C). Of particular interest is that the evaluated specimens from Santa Fe District in northern Veraguas correspond with a darker chromatic variation of *R. pallescens* infected by specific genetic groups of *T. rangeli* and *T. cruzi* [44]. Although the genetic characteristics of this population have not been studied, the reported phenotypic differences and the differences found in their microbial composition could be explained by the presence of this dark chromatic variant in this geographical region. However, it is not known if this dark variant represents a separate geographical population, a new subspecies, or a new distinct species of *R. pallescens*.

Geographical differences in microbiota have been observed in ticks [15, 18, 20, 24, 25], but not in mosquitoes where species-specific microbiota is thought to be stable [36]. One study in triatomines did not observe any difference in the microbiota between three distant locations in the southern USA for *Triatoma protracta* [23], which is a trend that has been confirmed [81] and opposed [82] in other hemipterans. As in other insect species, taxonomic clade-wide stability of microbiota composition with regard to one environmental variable or another does not appear to be consistent. Generalizations about geographical variation in insect microbiomes will have to remain at the species level for now. However, this is still very much an open question in triatomine microbiota research.

Contrary to our expectations, there was no significant difference in bacterial community composition between *T. cruzi*-infected and uninfected individuals. This contrasts with previous studies that have found significant differences between microbiomes of *T. cruzi*-positive and negative individuals [23, 30]. However, our small sample size, limited number of palms sampled (*n* = 8) with skewed infection ratios, and a skewed abundance of younger stage nymphs (N1–N3), may confound true observable differences. Furthermore, detectable levels of differences may be localized to a portion of the triatomine gut where *T. cruzi* develops; this deserves further study and experimental controls. A similar situation may occur during infection with *T. rangeli*, which can colonize not only the insect intestine but also the hemocoel and salivary glands [83]. We also did not find any significant difference in the microbiota as a result of the dominant blood-meal source found, as previously observed [31]. This could be due to the complexity of variables that influence the microbiota. While blood-meal source potentially has an effect on the bacterial composition in the gut, these samples often have mixed blood-meal sources with differing abundance (Additional file 1: Table S1; [50]), making discrete differences difficult to observe. Habitat associated microbial composition was found to be different between peridomestic and peridomestic-forest; however, this observation represents a few peridomestic-forest samples from northern Veraguas only and likely is an artifact of location difference. Similarly, microbial composition between age classes showed no differences, but because this dataset is highly skewed toward a couple of nymphal classes, distinctions are impossible to detect. This study examined the whole-body microbiota, which may obscure anatomically localized differences observed in other studies and, on the whole, result in a more comprehensive measure of microbial composition where local environment has a bigger impact.

## Conclusions

In conclusion, we examined the whole-body microbiota of *Rhodnius pallescens*, which can serve as a benchmark for future comparative studies examining the microbiota of specific organs or anatomical regions. Interestingly, the largest difference in *R. pallescens* microbial community composition was between geographical locations. While we did not find any definitive differences in relation to other variables (e.g. habitat type, age class, blood-meal source, infection status) these remain important aspects of vector biology that require further study. The effects of geographical environmental diversity can be minimized through the use of more comparative studies using laboratory-reared insects and controlled studies to tease apart more complex variables such as blood-meal sources and infection status.

## Supplementary information

**Additional file 1: Table S1.** Metadata for all samples used in this study including NCBI accession numbers.

**Additional file 2: Table S2.** Phylum level read counts and proportions for each of the three locations.

**Additional file 3: Figure S1.** Heat-trees showing the abundance of reads for each taxonomic group (branches) across the three collection localities.

**Additional file 4: Table S3.** Top 20 Family level read counts and proportions for each of the three locations.

**Additional file 5: Table S4.** Top 20 Genus level read counts and proportions for each of the three locations.

**Additional file 6: Table S5.** Total number reads and the number and proportion of Wolbachia reads for each sample across all locations.

**Additional file 7: Figure S2.** Alpha richness plot across locations.

**Additional file 8: Figure S3.** Alpha richness plot across habitat type.

**Additional file 9: Figure S4.** Alpha richness plot across infection status.

**Additional file 10: Figure S5.** Non-metric multidimensional scaling plot (based on Bray-Curtis distances) of OTU frequency for the microbial communities of triatomines across pastures in Veraguas.

**Additional file 11: Figure S6.** Non-metric multidimensional scaling plot (based on Bray-Curtis distances) of OTU frequency for the microbial communities of triatomines across location at peridomestic sites with (A) and without Veraguas (B) shown.

**Additional file 12: Figure S7.** Non-metric multidimensional scaling plot (based on Bray-Curtis distances) of OTU frequency for the microbial communities of triatomines across palm type at Veraguas sites.

**Additional file 13: Figure S8.** Non-metric multidimensional scaling plot (based on Bray-Curtis distances) of OTU frequency for the microbial communities of triatomines across palm type at Las Pavas sites.

**Additional file 14: Figure S9.** Non-metric multidimensional scaling plot (based on Bray-Curtis distances) of OTU frequency for the microbial communities of triatomines across Infection status at Las Pavas sites.

**Additional file 15: Figure S10.** Non-metric multidimensional scaling plot (based on Bray-Curtis distances) of OTU frequency for the microbial communities of triatomines across infection status at peridomestic sites.

**Additional file 16: Figure S11.** Non-metric multidimensional scaling plot (based on Bray-Curtis distances) of OTU frequency for the microbial communities of triatomines across infection status at Trinidad de las Minas sites.

**Additional file 17: Figure S12.** Non-metric multidimensional scaling plot (based on Bray-Curtis distances) of OTU frequency for the microbial communities of triatomines across infection status at Veraguas sites.

**Additional file 18: Figure S13.** Multivariate Principal Coordinate Analyses results showing location by age class (**a**) and age class by location (**b**).

**Additional file 19: Table S6.** Total read counts and proportions for all observed bacterial taxa down to the genus level for each of the three sampling locations.

## Data Availability

Data supporting the conclusions of this article are included within the article and its additional files. Demultiplexed reads are available from NCBI Sequence Read Archive under BioProject ID PRJNA543522 and Submission ID SUB5631094. Metadata and NCBI accession numbers are in Additional file 1: Table S1.
