# Supplementary figures and images for: Regional biogeography of microbiota composition in the Chagas disease vector Rhodnius pallescens
Source: Parasit Vectors. 2019 Oct 29;12:504. doi: 10.1186/s13071-019-3761-8 (PMC6821009; doi:10.1186/s13071-019-3761-8)

1. Trinidad de las Minas

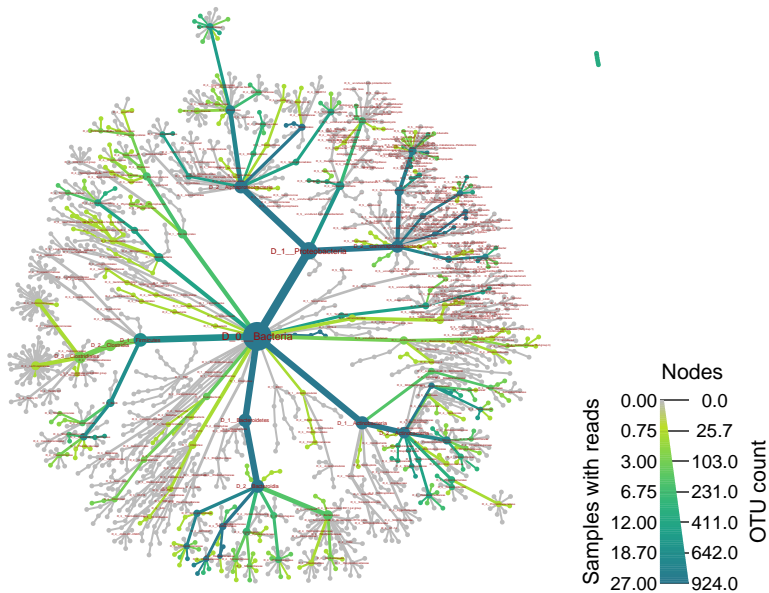

2. Las Pavas

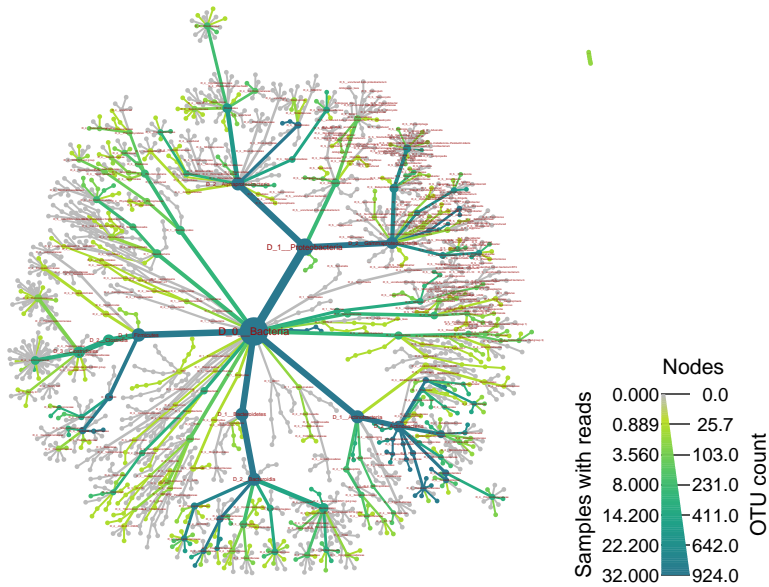

3. Veruaguas

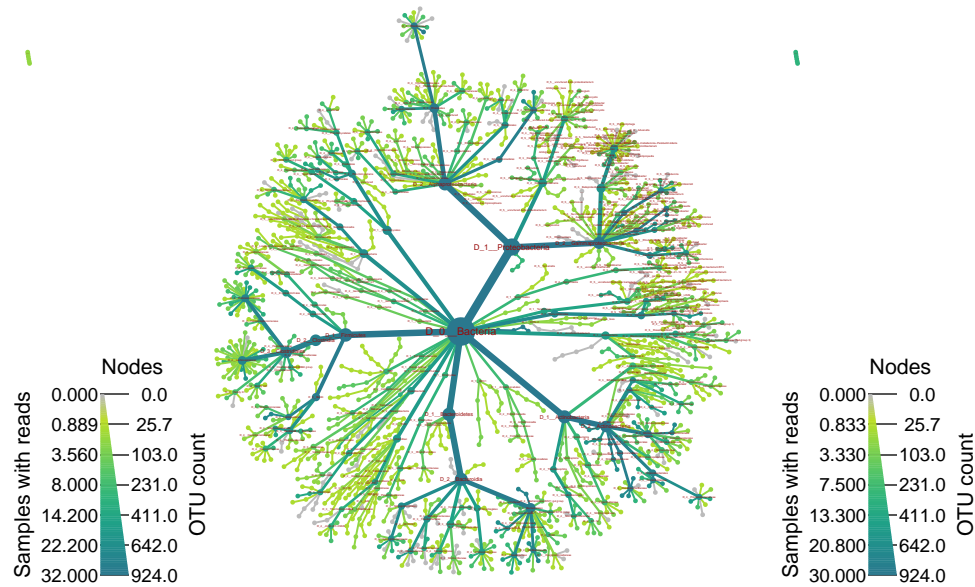

Supplement: Supplementary file 3 — Additional file 3: Figure S1. Heat-trees showing the abundance of reads for each taxonomic group (branches) across the three collection localities. [file 13071_2019_3761_MOESM3_ESM.pdf]

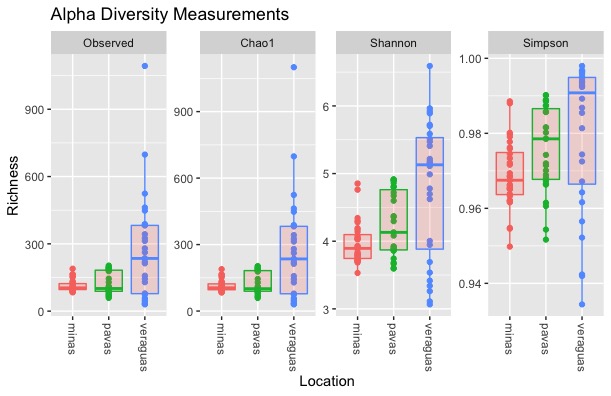

Supplement: Supplementary file 7 — Additional file 7: Figure S2. Alpha richness plot across locations. [file 13071_2019_3761_MOESM7_ESM.jpeg]

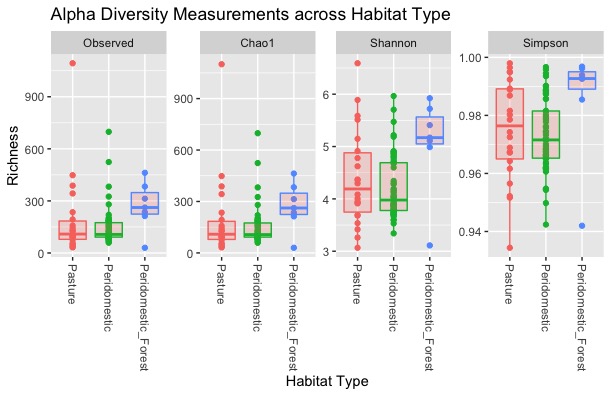

Supplement: Supplementary file 8 — Additional file 8: Figure S3. Alpha richness plot across habitat type. [file 13071_2019_3761_MOESM8_ESM.jpeg]

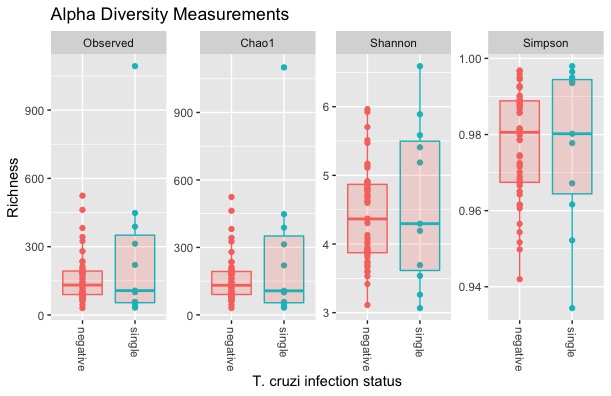

Supplement: Supplementary file 9 — Additional file 9: Figure S4. Alpha richness plot across infection status. [file 13071_2019_3761_MOESM9_ESM.jpeg]

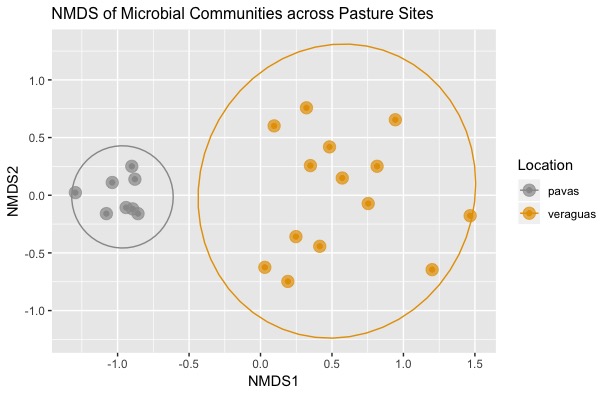

Supplement: Supplementary file 10 — Additional file 10: Figure S5. Non-metric multidimensional scaling plot (based on Bray-Curtis distances) of OTU frequency for the microbial communities of triatomines across pastures in Veraguas. [file 13071_2019_3761_MOESM10_ESM.jpeg]

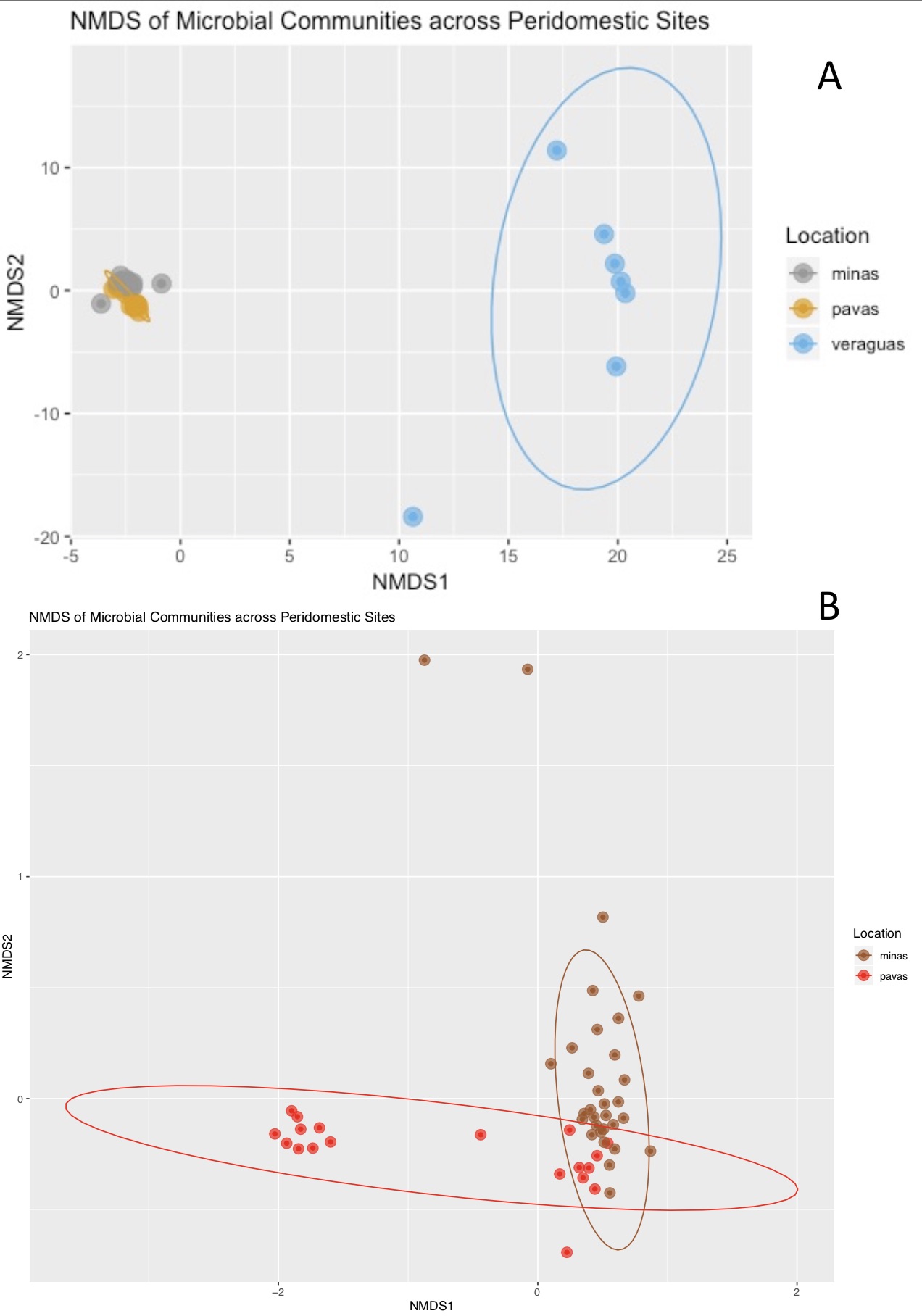

Supplement: Supplementary file 11 — Additional file 11: Figure S6. Non-metric multidimensional scaling plot (based on Bray-Curtis distances) of OTU frequency for the microbial communities of triatomines across location at peridomestic sites with (A) and without Veraguas (B) shown. [file 13071_2019_3761_MOESM11_ESM.jpg]

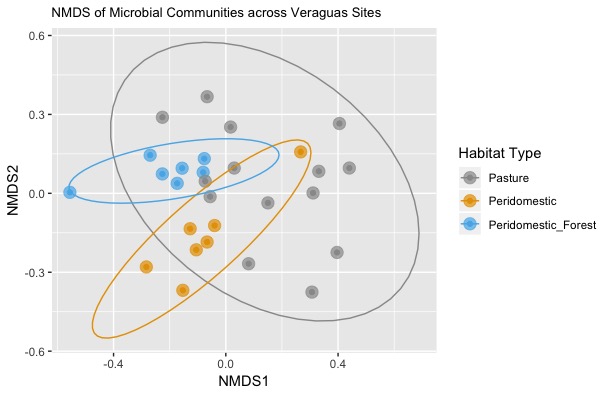

Supplement: Supplementary file 12 — Additional file 12: Figure S7. Non-metric multidimensional scaling plot (based on Bray-Curtis distances) of OTU frequency for the microbial communities of triatomines across palm type at Veraguas sites. [file 13071_2019_3761_MOESM12_ESM.jpeg]

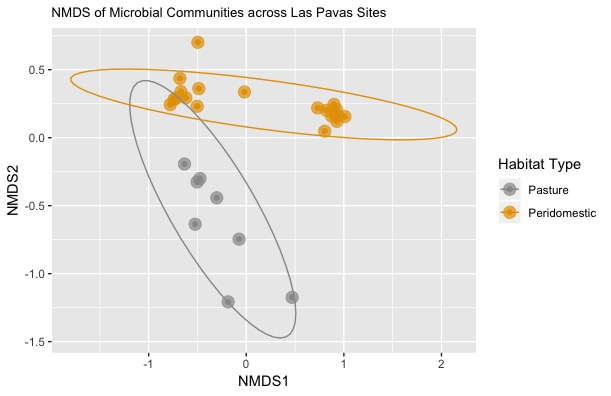

Supplement: Supplementary file 13 — Additional file 13: Figure S8. Non-metric multidimensional scaling plot (based on Bray-Curtis distances) of OTU frequency for the microbial communities of triatomines across palm type at Las Pavas sites. [file 13071_2019_3761_MOESM13_ESM.jpeg]

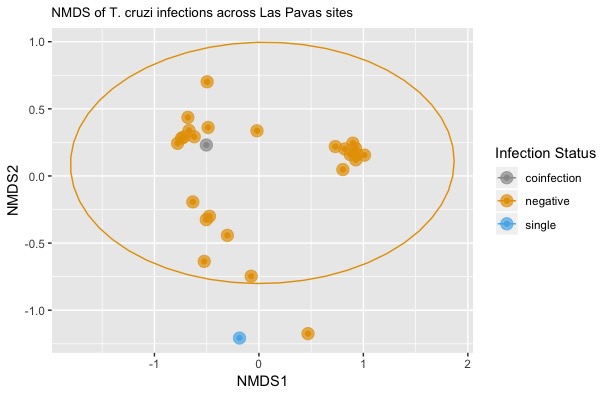

Supplement: Supplementary file 14 — Additional file 14: Figure S9. Non-metric multidimensional scaling plot (based on Bray-Curtis distances) of OTU frequency for the microbial communities of triatomines across Infection status at Las Pavas sites. [file 13071_2019_3761_MOESM14_ESM.jpeg]

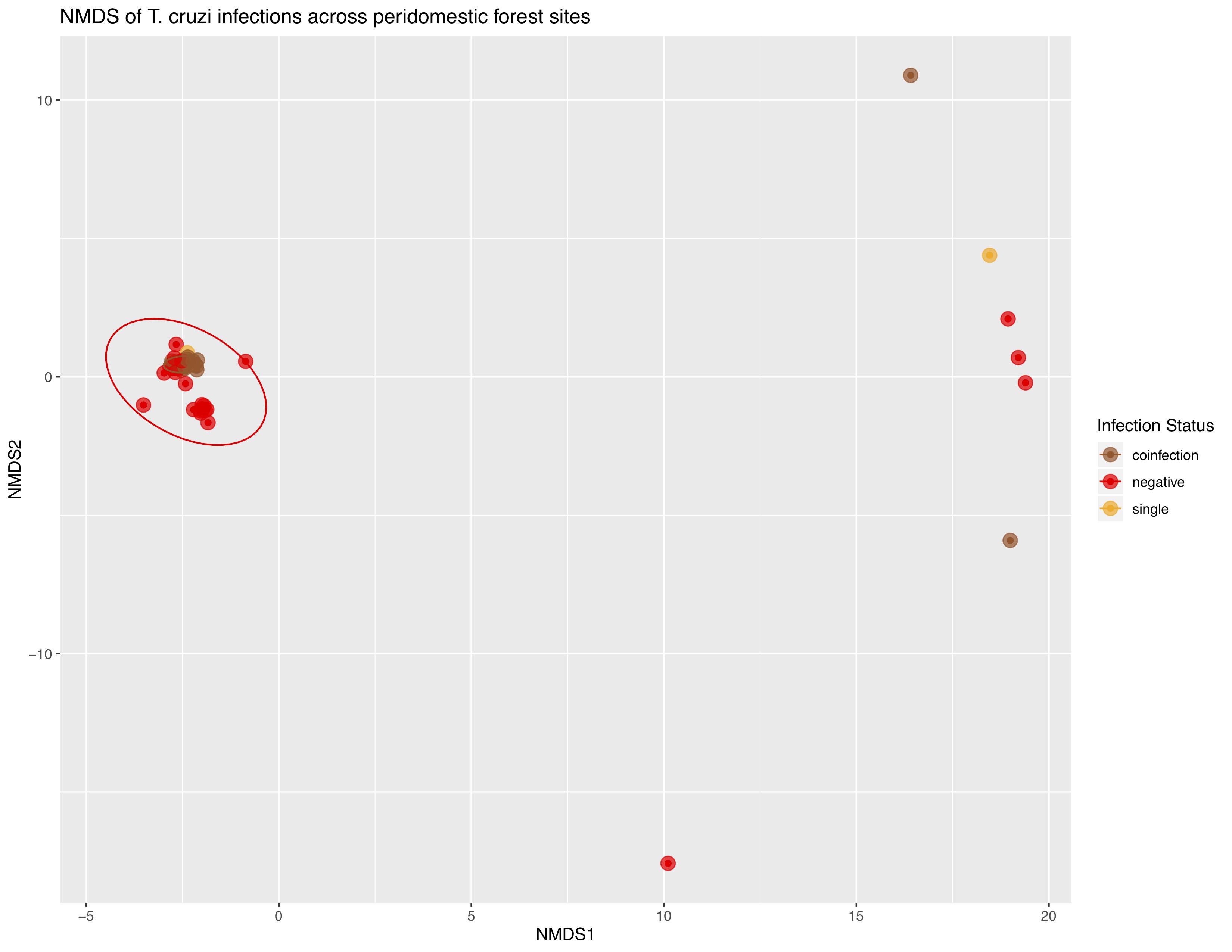

Supplement: Supplementary file 15 — Additional file 15: Figure S10. Non-metric multidimensional scaling plot (based on Bray-Curtis distances) of OTU frequency for the microbial communities of triatomines across infection status at peridomestic sites. [file 13071_2019_3761_MOESM15_ESM.jpg]

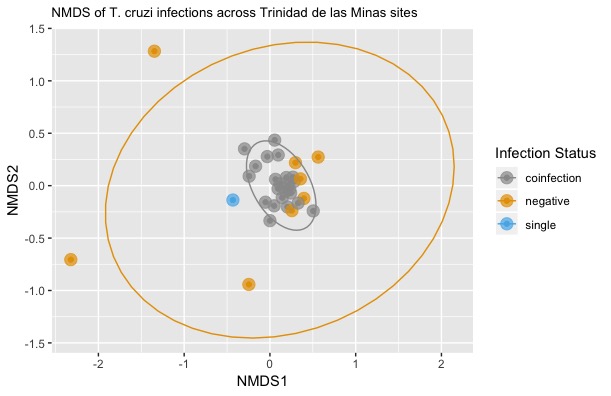

Supplement: Supplementary file 16 — Additional file 16: Figure S11. Non-metric multidimensional scaling plot (based on Bray-Curtis distances) of OTU frequency for the microbial communities of triatomines across infection status at Trinidad de las Minas sites. [file 13071_2019_3761_MOESM16_ESM.jpeg]

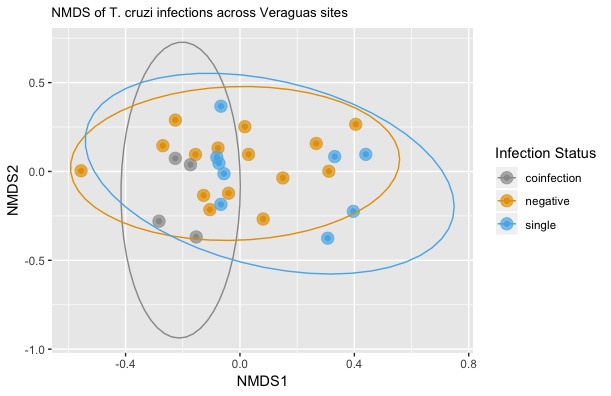

Supplement: Supplementary file 17 — Additional file 17: Figure S12. Non-metric multidimensional scaling plot (based on Bray-Curtis distances) of OTU frequency for the microbial communities of triatomines across infection status at Veraguas sites. [file 13071_2019_3761_MOESM17_ESM.jpeg]

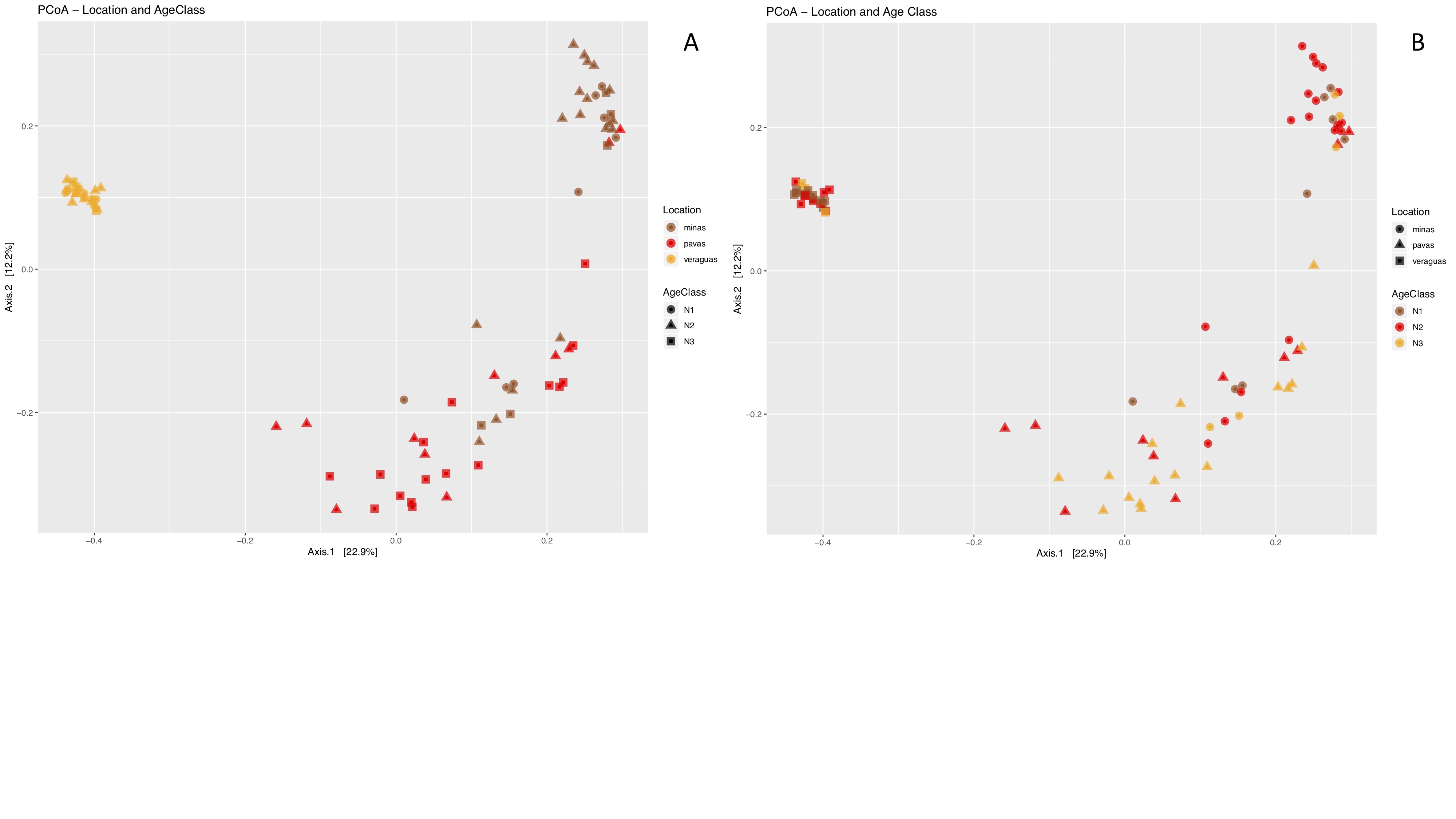

Supplement: Supplementary file 18 — Additional file 18: Figure S13. Multivariate Principal Coordinate Analyses results showing location by age class (a) and age class by location (b). [file 13071_2019_3761_MOESM18_ESM.jpg]
